# Supplementary material for: Biochemical and Hematological Markers in Workers with Chronical Exposure to Lead and Cadmium in Colombia
Source: Toxics. 2022 Sep 1;10(9):524. doi: 10.3390/toxics10090524 (PMC9502882; doi:10.3390/toxics10090524)
Supplement: Supplementary file 1 [file toxics-10-00524-s001.zip › toxics-1828480-supplementary.pdf]

Supplementary Materials: Biochemical and Hematological Markers in Workers with Chronical Exposure to Lead and Cadmium in Colombia

Fredy Vergara-Murillo, Katiana Martinez-Yanez, Álvaro Fortich-Revollo, Ángel Paternina-Caicedo and Boris Johnson-Restrepo

Method for determination of Lead and Cadmium in whole blood.

For the present study, whole blood was used, collected in 4 mL tubes with ethylenediaminetetraacetic acid (EDTA), the standard added Cd and Pb analysis was used, using the matrix modifier magnesium nitrate (Merck®, Darmstadt, Germany) and ammonium dihydrogen phosphate (Merck®, Darmstadt, Germany), respectively, Triton X (Merck) and 0.02% nitric acid (Merck®, Darmstadt, Germany) was used as a diluent and (Table 1). We used three concentrations of goat blood-based standard reference materials (CRMs) provided by the New York State Department of Health (Wadsworth Center, Albany, NY, US)[35].

The analytical method used to determine Pb and Cd in whole blood samples were based upon an Atomic Absorption Spectrometer (AAS) iCE 3500 series equipped with a graphite furnace atomizer, Zeeman-effect-background correction, and auto-sampler (Thermo Fisher Scientific, Cambridge, UK). All samples were analyzed in duplicate, the carrier gas was argon, the injection volume was 20 μL and the value of the area under the curve was used to determine the measurements and metal concentrations. Before each analysis, the calibration curve was performed with human blood by the aggregate standard method, and for quality control, measurements of three CRM were performed in each run. A reagent blank (all reagents mixed in one container with EDTA, no whole blood sample) and an air sample were also read each day. All the calibration curves complied with an R^2 greater than 0.995 and the CRM within the reference values to proceed with the reading of the samples, the reading of whole blood samples was performed; every 10 samples an SRM was included to verify the quality during the analysis. Whole blood samples that had above-recommended concentrations of Cd and Pb were given a second reading to verify. For the calibration and validation of the method, the coefficient of variation and dispersion was reported from three experiments with SRM: BE 12-11, BE 12-13 and BE 12-14 for determination of Pb, and from three experiments with BE 12-12, BE 12-14 and BE 12-15 for Cd analysis.

Table S1. Parameters of the analysis by atomic absorption (graphite furnace).

Metal	Wavelength	Lamp Current	Standards concentration.
**Cd**	228.8nm	50%	1/2/3/4/6 (μg/L)
**Pb**	283.3 nm	90%	2.5/5/10/20/40/60 (μg/dL)

Table S2. Reproducibility of whole blood analysis and repeatability by atomic absorption spectrometry. Cd and Pb were determined by graphite furnace.

	**Cd**		**Pb**	
**Repeatability**	2.0%[a]	3.2%[b]	2.2%[c]	2.5%[d]
**Reproducibility**	5.1%[e]	4.5%[f]	3.5%[g]	2.0%[h]

[a] for the 2 μg/L Cd, standard (*n* = 8). [b] for the 3.2 μg/L Cd, CRM (BE 12-14) (*n* = 8). [c] for the 5 μg/dL Pb, standard (*n* = 8). [d] for the 4.2 μg/dL Pb, CRM (BE12-13) (*n* = 8). [e] for the 3 μg/L Cd, standard (*n* = 8). [f] for the 2.4 μg/L Cd, CRM (BE 12-12) (*n* = 8). [g]for the 10 μg/dL Pb, standard (n=8). [h] for the 8.4 μg/dL Pb, CRM (BE12-11) (*n* = 8).

These experiments of repeatability and reproducibility were made with standard added and standard reference materials (SRM).

Table S3. Temperature program of the graphite furnace atomic absorption instrument for Cd.

	Step	Temperature (°C)	Time (s)	Ramp (°C/s)	Gas flow (L/min)
1	Drying 01	110	50	10	0.1
2	Drying 02	200	5	10	0.1
3	Pyrolysis	420	15	150	0.1
4	Atomization	970	3	0	Off
5	Cleaning	2500	3	0	0.2

Table S4. Temperature program of the furnace atomic absorption spectrometer for Pb.

	Step	Temperature (°C)	Time (s)	Ramp (°C/s)	Gas Flow (L/min)
1	Drying 01	110	50	10	0.1
2	Drying 02	200	5	10	0.1
3	Pyrolysis	850	20	150	0.2
4	Atomization	1600	3	0	off
5	Cleaning	2500	3	0	0.2

Table S5. Limits of detection and quantification in whole blood analysis by atomic absorption spectrometry.

Metal	MDL	MQL
**Cd, μg/L**	0.31	1.03
**Pb, μg/dL**	0.41	1.36

*MDL: method detection limit; MQL: method quantification limit.

Table S6. Accuracy of the analysis of certified reference materials for whole blood expressed as percentage recovery.

	CRM	Expected concentration	Concentration found	Recovery %	RDS	Reproducibility %
**Pb, μg/dL**	BE12-11	8.4	9.32± 1.0	111	0,014	3.2
	BE12-13	4.2	4.56 ± 0.5	108	0,09	5.2
	BE 12-14	25.4	25.7 ± 0.7	108	0,027	5.5
**Cd, μg/L**	BE 12-12	2.4	2.32 ± 0.2	97	0,08	6.7
	BE 12-14	3.2	2.8 ± 0.3	87	0,068	3.4
	BE 12-15	11.3	12.37± 0.6	109	0,048	4.2

Note: Quality control was performed with goat whole blood certificated reference material (CRM) provided by the Department of Health of New York State (Wadsworth Center, Albany, NY, US). Percent recovery is the measure of accuracy of the concentrations obtained in the analyzes compared with the concentrations reported by the author. Accuracy is expressed in percentage of reproducibility, expressing the percentage change between measurements of the same sample.
